# Sediment‐associated organic matter sources and sediment oxygen demand in a Special Area of Conservation (SAC): A case study of the River Axe, UK

**DOI:** 10.1002/rra.3175

**Published:** 2017-06-29

**Authors:** A. L. Collins, Y. Zhang, S. McMillan, E. R. Dixon, A. Stringfellow, S. Bateman, D. A. Sear

**Affiliations:** ^1^ Rothamsted Research Okehampton UK; ^2^ RSK ADAS UK Ltd. Stratford‐upon‐Avon UK; ^3^ Civil Engineering and Environment University of Southampton, Highfield Campus Southampton UK; ^4^ Geography and Environment University of Southampton, Highfield Campus Southampton UK

**Keywords:** fingerprinting, sediment‐associated organic matter, sediment oxygen demand, uncertainty

## Abstract

Oxygen demand in river substrates providing important habitats for the early life stages of aquatic ecology, including lithophilous fish, can arise due to the oxidation of sediment‐associated organic matter. Oxygen depletion associated with this component of river biogeochemical cycling, will, in part, depend on the sources of such material. A reconnaissance survey was therefore undertaken to assess the relative contributions from bed sediment‐associated organic matter sources potentially impacting on the River Axe Special Area of Conservation (SAC), in SW England. Source fingerprinting, including Monte Carlo uncertainty analysis, suggested that the relative frequency‐weighted average median source contributions ranged between 19% (uncertainty range 0–82%) and 64% (uncertainty range 0–99%) for farmyard manures or slurries, 4% (uncertainty range 0–49%) and 35% (uncertainty range 0–100%) for damaged road verges, 2% (uncertainty range 0–100%) and 68% (uncertainty range 0–100%) for decaying instream vegetation, and 2% (full uncertainty range 0–15%) and 6% (uncertainty range 0–48%) for human septic waste. A reconnaissance survey of sediment oxygen demand (SOD) along the channel designated as a SAC yielded a mean SOD_5_ of 4 mg O_2_ g^−1^ dry sediment and a corresponding SOD_20_ of 7 mg O_2_ g^−1^ dry sediment, compared with respective ranges of 1–15 and 2–30 mg O_2_ g^−1^ dry sediment, measured by the authors for a range of river types across the UK. The findings of the reconnaissance survey were used in an agency (SW region) catchment appraisal exercise for informing targeted management to help protect the SAC.

## 1 INTRODUCTION

There is a clear requirement for improved catchment management strategies aimed at controlling fine‐grained sediment and associated organic matter (OM) mobilization and delivery to watercourses to help support the maintenance of good water quality and ecological status. Well‐documented specific impacts of excess sediment as a stressor on aquatic ecology include gill clogging, histological changes, reduced resistance to disease, suppressed feeding efficiency, and the smothering of progeny incubating in fish spawning gravels (Greig, Sear, Smallman, & Carling, 2005; Kemp, Sear, Collins, Naden, & Jones, 2011). Sediment‐borne OM entering rivers and ingressing the benthic zone competes with aquatic ecology for the supply of dissolved oxygen, by imparting a demand during decay and oxidation, and thereby hampers survival of a range of species (Chevalier, Carson, & Miller, 1984; Greig et al*.*, 2005). To date, however, this component of sediment has received less attention even though its improved management has recently been underscored as important for helping the UK to achieve environmental objectives around aquatic ecological status (Collins et al*.*, 2011; Sear et al*.*, 2014, 2016).

The management of the aquatic environment in the UK has, to a large extent, been underpinned by a three‐pronged approach comprising targeted advice for farmers and incentives for mitigation methods such as agri‐environment measures and environmental regulation. Improved management of the diffuse pollution problem, including the detrimental impacts of sediment‐associated OM on aquatic ecology, requires mitigation strategies to be underpinned by a catchment‐wide perspective on the key sources of the problem. This is essential because the off‐site impacts experienced in river habitats, including spawning gravels, reflect distributed (diffuse and point) inputs from across upstream landscapes. Applying traditional sediment measurement and monitoring techniques on a spatially distributed basis face many logistical problems and issues of cost, and as a result, sediment fingerprinting procedures have been increasingly used to document key sources at catchment scale (Collins et al*.*, in press; Walling, 2013; Walling & Foster, 2016). Where information is required in a short timescale to start informing management decisions, the source fingerprinting approach has proved useful in catchment reconnaissance surveys (e.g., Walling, Collins, & McMellin, 2003). At the same time, some recent studies have demonstrated the utility of sediment source tracing procedures to apportion inputs from catchment sources of sediment‐associated OM, with a view to supporting more holistic management of the sediment problem in the UK (Collins et al*.*, 2014; Cooper et al*.*, 2015).

Against the above background, a reconnaissance study was undertaken to provide new evidence for the management of the River Axe Special Area of Conservation (SAC), UK. It was intended that the new work should build upon the evidence base provided by a recent source‐tracing study focussing on the primary sources of minerogenic bed sediment silting salmonid spawning gravel habitats along the River Axe (Collins et al*.*, 2012). Accordingly, the new work examined the provenance of sediment‐associated OM and undertook sediment oxygen demand (SOD) measurements to ensure that the evidence base underpinning catchment management decisions for sediment control takes account of the key process linkages degrading the quality of the aquatic environment for ecology.

## 2 THE RIVER AXE SAC AND UPSTREAM STUDY AREAS

**Figure 1:**
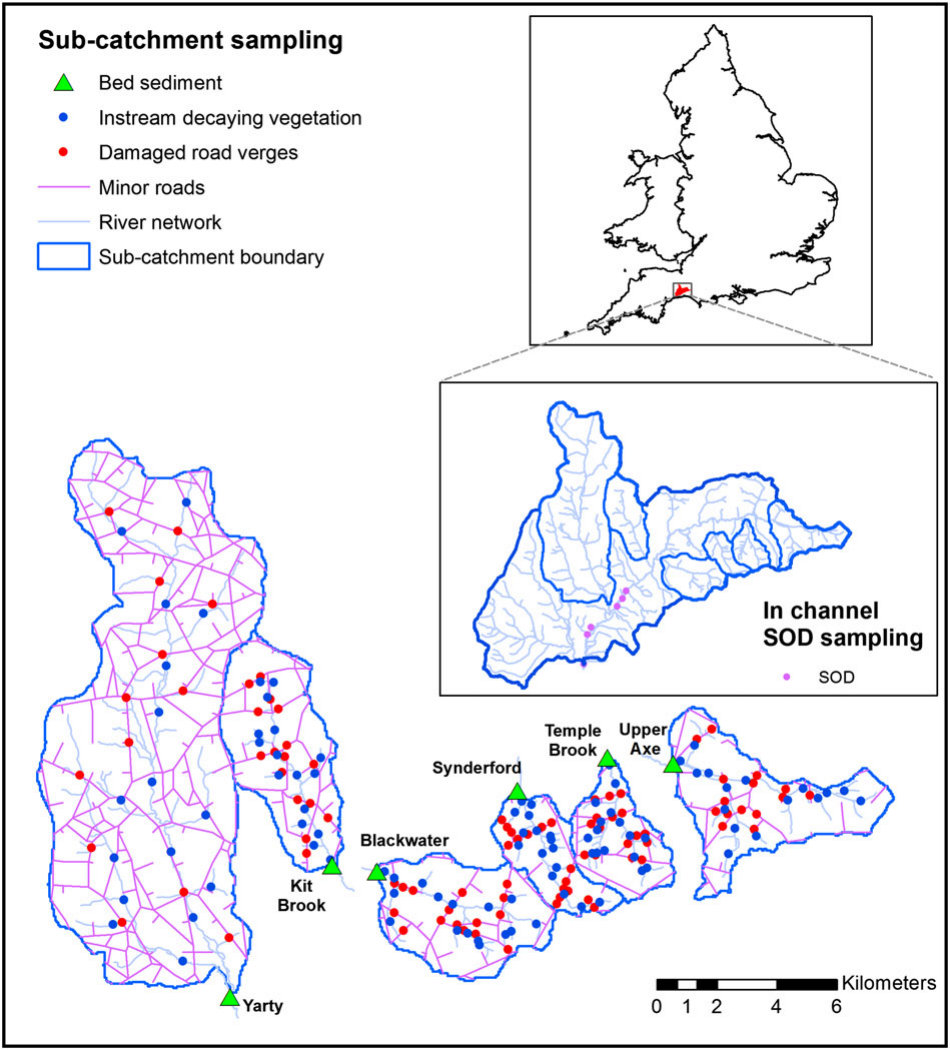
The River Axe catchment, showing the channel bed sediment sampling locations for the source tracing and sediment oxygen demand (SOD) work, plus the locations from which composite source samples were collected to represent the damaged road verge and instream decaying vegetation source categories. The terms of the funding contract did not permit the locations of farm manure or slurry or domestic septic tank sampling to be disclosed given the sensitivities around these potential point sources. Here, it is important to note that adherence to this condition was frequently a prerequisite for the field team gaining access to sampling sites [Colour figure can be viewed at wileyonlinelibrary.com]

The River Axe (Figure 1).SAC extends for 13 km downstream from the confluence with the Blackwater River, meandering through a well‐developed floodplain dominated by improved dairy pasture. The SAC is designated for watercourses of plain to montane levels with crowfoots (*Ranunculion fluitantis*) and starworts (*Callitricho‐Batrachion*) vegetation, sea lamprey (Petromyzon marinus), brook lamprey (Lampetra planeri), and bullhead (Cottus gobio). The River Axe SAC is also notified as a Site of Special Scientific Interest (SSSI) for its nationally important geomorphology (which demonstrates contrasting patterns of meander formation), for the presence of otter (Lutra lutra) and medicinal leech (Hirudo medicinalis), and for its diverse invertebrate communities. An Atlantic salmon (Salmo salar) run was extirpated in the 1980s, and currently, the site is in “unfavourable condition.” This is believed to be for a variety of reasons including degraded water quality, primarily due to siltation and associated OM inputs. Natural England's objective for “favourable condition” in SSSIs/SACs designated for river habitat has been set out in Common Standards agreed by the UK conservation agencies in response to the European Union Habitats Directive. These Common Standards apply equally to SSSI and SAC designations and relate to the availability of the river (as a representative of its type) to provide favourable habitat conditions for the characteristic biological community, rather than conditions that might favour a particular individual species. Table 1 presents summary information for the study subcatchments.

**Table 1:** Background data for the River Axe subcatchments

FEH attribute	Upper River Axe	Temple Brook	River Synderford	Blackwater River	Kit Brook	River Yarty	Main channel River Axe SAC
Area (km^b^)^a^	20	10	9	18	20	95	304
Mean altitude (m)	150	146	152	129	163	139	134
Base flow index	0.526	0.547	0.48	0.398	0.617	0.399	0.499
Mean slope (m km^−1^)	80.3	88.7	106.6	91.7	93	99.6	90.5
Mean flood depth (cm)	0.397	0.187	0.087	0.094	0.072	0.328	0.457
Median annual maximum 1‐hr rainfall (mm)	11.6	11.6	11.7	11.5	11.4	11.3	11.4
Median annual maximum 1‐day rainfall (mm)	42.3	41.1	40.8	40.6	39.4	39.5	39.9
Average annual rainfall for period 1961–1990 (mm)	986	1,003	1,013	1,024	1,003	1,014	991
Standard percentage run‐off	37.95	39.09	44.31	43.49	32.12	41.98	38.73
% urban^b^	3	5	3	2	3	3	5
% water^b^	1	1	1	1	1	1	1
% woodland^b^	5	3	7	10	10	11	9
% rough grazing^b^	8	4	6	5	6	6	6
% arable^b^	12	15	16	12	24	12	14
% improved grazing^b^	71	72	67	70	56	67	65

*Note*. FEH = Flood Estimation Handbook version 3.0; SAC = Special Area of Conservation.

a Derived using the CatchmentsUK tool from Wallingford Hydro Solutions Ltd.

b Based on the ADAS land use database combining the CEH land cover map and the June Agricultural Survey Returns (see Comber, Anthony, & Proctor, 2008 for background).

### 2.1 The sediment‐associated OM fingerprinting methodology

#### 2.1.1 Field sampling

The collection (February 2013) of representative source material samples for fluvial bed sediment‐associated OM encompassed four potential source categories. These source types were farmyard manures or slurries, damaged road verges, decaying instream vegetation, and point source human septic waste discharges. The source type categories were finalized during discussions with Natural England. Table 2 summarizes the source sample numbers. In the case of the farm manures or slurries, damaged road verge, and decaying instream vegetation source categories, every 10 subsamples collected in the field were bulked into a composite sample for subsequent laboratory analysis. For the human septic waste source category, every five subsamples were bulked into a composite for laboratory analysis. The sampling of farmyard manures or slurries included fresh excreta from yards, steadings, and housings, as well as material from dry or wet stores and manure heaps in fields. Sampling included both cattle and sheep excreta. The random subsampling was designed to be spatially representative of each yard, steading, housing, store, or field heap in question. Damaged road verge samples were collected from 100‐m sections of degraded road margins selected randomly to be representative of the entire road network in each subcatchment and comprised the leaf litter and mulch present in degraded road margins damaged by vehicle and livestock traffic. Representative samples of decaying instream vegetation were retrieved along the channel system from specific 100‐m reaches selected randomly along the river network of each subcatchment and included material trapped by coarse woody debris and additional obstacles. Septic tank subsamples were collected from the chambers or drainage field biomat of each individual installation and bulked into a single composite. Locally, septic tanks were considered to be the primary source of spatially distributed risk for human septic waste reaching subcatchment watercourses as opposed to sewage treatment works. Information on the connectivity of sources to streams is provided in Supplementary Information (SI). All source samples were placed in cool boxes with ice packs immediately upon collection in the field and remained in such storage for transport to the laboratory (typically on the same day).

**Table 2:** Summary of the subsample numbers collected during the field campaign

	Sediment‐associated OM sources				Bed sediment for source apportionment		Bed sediment for SOD
Subcatchment	Farm manures or slurries	Damaged road verges	Decaying instream vegetation	Human septic waste point source discharges	Location (UK National Grid reference and latitude/longitude) of sample collection		
Upper River Axe	80	80	80	40	ST429059 50.849786 N −2.812477 W	18	
Temple Brook	80	80	80	40	ST409052 50.843291 N −2.840771 W	18	
River Synderford	80	80	80	40	ST377047 50.838459 N −2.886130 W	18	
Blackwater River	80	80	80	40	ST330021 50.814556 N −2.952392 W	18	
Kit Brook	80	80	80	40	ST315024 50.817077 N −2.973737 W	18	
River Yarty	80	80	80	40	ST281982 51.677944 N −3.041269 W	18	
Main channel River Axe SAC^a^	‐	‐	‐	‐	ST324023 To SY259927 50.816284 N −2.960944 W to 50.729173 N −3.051258	‐	24

*Note*. OM = organic matter; SOD = sediment oxygen demand; SAC = Special Area of Conservation.

The ‘‐’ is used to indicate no samples were collected.

a Samples collected along the main channel reach designated as the SAC.

Representative samples of fine‐grained channel bed sediment for both the sourcing exercise and for assessing SOD were collected during February 2013 using an established remobilization technique (Collins et al*.*, 2012; Duerdoth et al*.*, 2015; Lambert & Walling, 1988). During sample collection, a metal stilling well (height 1.1 m and surface area 0.18 m^2^) was carefully lowered onto, and pushed into, the river bed to provide a means of minimizing the loss of remobilized sediment by winnowing. The river water and upper 10–20 cm of the channel bed enclosed in the stilling well were stirred and agitated using a portable battery‐powered drill equipped with a plaster stirrer fitting. Agitation of both the water column and river bed provided a basis for sampling fine‐grained sediment stored both as a surface drape and within the interstices of the bed matrix. Each bed sediment sample (total volume of 1 L) comprised a composite of two subsamples (~0.5 L each) retrieved from different points in the channel at each location. More detail is provided in SI. Three composite samples were collected in triplicate at each channel location in conjunction with the source tracing exercise (Figure 1; Table 2). For SOD measurements, two composite samples were collected from each channel location along the SAC (Figure 1; Table 2). The river water and substrate were consistently agitated for 60 s prior to the depth‐integrated sampling of the remobilized sediment within the stilling well. Collection of sediment to a depth of 10–20 cm helped to ensure retrieval of deposited and ingressed material from the layer of the river substrate used by a variety of species but especially lithophilous fish. All river channel bed sediment samples were returned to laboratory fridge storage on the day of sampling in acid‐washed polyethylene containers transported in a cool box with ice packs. Samples were de‐watered using settling and decanting.

#### 2.1.2 Laboratory analyses

The source material and bed sediment samples collected for the sourcing work were wet sieved using a cascade of two apertures (250 and 63 μm) and subsequently oven dried at 40°C. Both the source material and sediment samples were ground to a fine powder prior to analysis for % total organic carbon and total nitrogen and stable isotope determinations of δ^13^C and δ^15^N values using a Carlo Erba NA2000 analyser (CE Instruments, Wigan, UK) and a SerCon 20‐22 isotope ratio mass spectrometer (SerCon Ltd., Crewe, UK). Wheat flour (1.91% N, 41.81% C, 4.8 δ^15^N, and −26.4 δ^13^C) calibrated against IAEA‐N‐1 by Iso‐Analytical, Crewe, UK, was used as a reference standard. The natural abundance stable isotope values were expressed using the standard δ notation with respect to the reference materials. Near infrared spectra were measured using an online Thermo Scientific Antaris 1 analyser that provided a spectral range of 4,000–10,000 cm^−1^ and returned 32 scans per sample with a typical resolution of 8 cm^−1^. All samples were run in triplicate with the average of these repeat runs used in subsequent statistical and numerical modelling data processing. No reagents were used in conjunction with the near infrared analyses.

The river channel bed sediment samples collected for SOD measurements were wet sieved using river water, and the <63‐μm fraction retained, as previous experience has shown that the highest SOD corresponds to this grain size fraction (Bateman, 2012). Duplicate 200‐ml aliquots of the wet‐sieved slurry were subsampled and transferred into 1‐L amber Duran flasks. When all of the bed sediment samples were processed, the flasks were placed in a Gallenkamp temperature‐controlled orbital incubator set to the average water temperature for the sample river. A shaker speed of circa 100 rpm was used to ensure adequate mixing of the sediment samples during the experiment and to mitigate the formation of oxygen gradients within the liquid. The flasks were sealed with tops in which were inserted a calibrated Q‐OX MediceL oxygen sensor (Shawcity Technology Ltd., error <1% signal) connected to a Delta‐T2 data logger. The flask contents were allowed to acclimate to operating conditions (approximately 1 hr) prior to commencing data logging. Oxygen concentration in the bottle headspace was sampled at 1‐min intervals and logged every 10 min for the 25‐day duration of the experiment. In order to correct for each individual oxygen probe, a blank experiment was run for 25 days using 200 ml of ultra‐high pure water. At the end of the 25‐day measurement period, the dry weight equivalent of the sediment in each test vessel was determined by filtering the slurry through dried, preweighed glass fibre/circle (GF/C) filter papers. The filters were subsequently oven dried for 2 hr at 100°C, cooled in desiccators, and weighed until constant values were achieved.

The output from the Q‐OX MediceL probes was converted into a mass of oxygen in the headspace. The sediment oxygen consumption (SOC) rate was calculated from the mass of oxygen consumed in the flask over time:

(1) $$\mathrm{SOC}=\frac{\left(m_{1}-m_{2}\right)}{t},$$

where SOC is the sediment oxygen consumption rate in mg O_2_ day^−1^, *t* is time in days, and *m*_1_ and *m*_2_ are the mass of oxygen at time 1 and time 2 in the headspace. All values were blank corrected. The final values were corrected using a Q10 (or Van't Hoff) equation to normalize the SOC estimates to 20°C:

(2) $${\mathrm{SOC}}^{20}=1.065^{\left(20‐T\right)}\cdot {\mathrm{SOC}}^{T},$$

where SOC^20^ is the rate at 20°C and *T* is the water temperature during measurement in degrees Celsius (Doyle & Lynch, 2005; Thomann & Mueller, 1987). This correction applies to temperatures of 10°C or more, which was the incubation temperature used in the experiments. The resulting rates were used to calculate the total mass of oxygen consumed over 0–5 days (SOC_5_) and 0–20 days (SOC_20_), respectively. These values were divided by the dry mass of sediment used in each flask to give SOD values in mg O_2_ g^−1^ dry sediment. Five days is a standard time period used to observe the oxygen demand of organic effluents and sewage wastes on water. A 25‐day time period is considered by convention to be an adequate time for the complete biochemical oxidation of organic material within water samples, sometimes known as the total biological oxygen demand (Delzer & McKenzie, 2003). It was considered that the 5**‐** and 20**‐**day periods used in the measurement of SOD are significant to incubating fish embryos because these rates provide a more accurate account of oxygen consumption by channel bed sediment over the embryo incubation period. It is critical to understand the longer term SOC of river channel bed sediments given the propensity for sediment retention in UK lowland rivers (e.g., Naden et al*.*, 2016) and the need to take into account the oxygen demand imparted by deposited sediment‐associated OM (Sear, Frostick, Rollinson, & Lisle, 2008).

#### 2.1.3 Data processing for source discrimination

**Figure 2:**
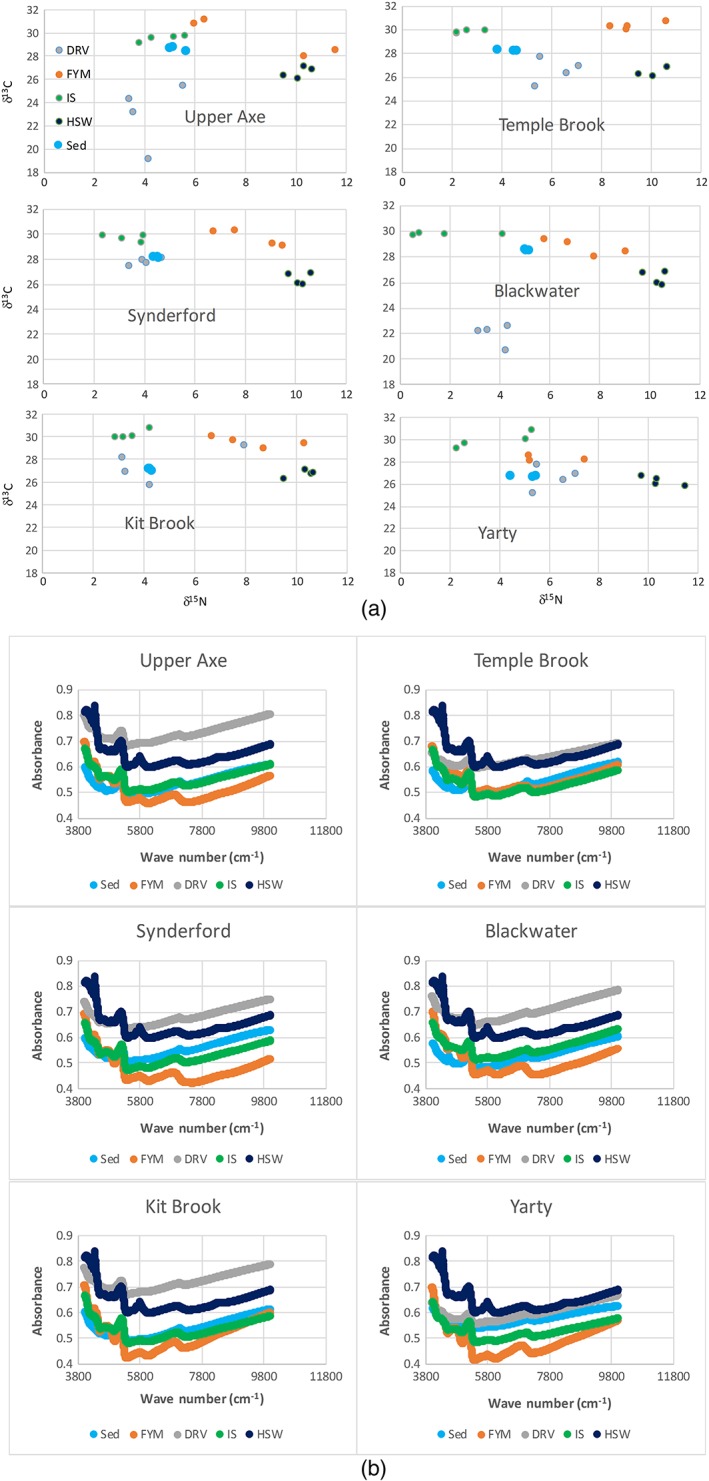
Plots comparing the subcatchment source and sediment sample bulk isotope data (panel a) and near infrared (panel b) spectra (FYM = farmyard manures or slurries; DRV = damaged road verges; IS = decaying instream vegetation; HSW = human septic waste) [Colour figure can be viewed at wileyonlinelibrary.com]

The data processing procedure is described in detail in Collins et al. (2014). The ranges (minimum and maximum) of the fingerprint property values measured for each source category were used to define parameter space for a mass conservation or bracket test (Figure 2) and only those properties for which the river channel bed sediment sample ranges were located in the mixing polygon represented by the OM source samples from the same subcatchment (Table 3) were entered into the statistical analysis for source discrimination.

**Table 3:** Fingerprint properties passing the mass conservation test

Upper River Axe	Temple Brook	River Synderford	Blackwater River	Kit Brook	River Yarty
ArNH_2_	ArNH_2_	ArNH_2_	ArNH_2_	ArNH_2_	ArNH_2_
ArOH	Aromatic	ArOH	ArOH	ArOH	ArOH
Aromatic	CH	Aromatic	Aromatic	Aromatic	Aromatic
Aromatic	CH_2_	C=H	C=H	C=H	C=H
CH	HC=CH	Cellulose	Cellulose	Cellulose	Cellulose
CH_2_	Protein	CH	CH	CH	CH
CH_3_	RNH_2_	CH_2_	CH_2_	CH_2_	CH_2_
CONH_2_	ROH	CH_3_	CH_3_	CH_3_	CH_3_
CONHR	Starch, glucose	CONH	CONH_2_	CONH	CONH_2_
HC=CH	δ^15^N	CONH_2_	CONHR	CONH_2_	CONHR
Protein		CONHR	HC=CH	CONHR	H_2_O
RNH_2_		H_2_O	Protein	HC=CH	HC=CH
ROH		HC=CH	RNH_2_	Protein	Protein
Starch		Protein	ROH	RNH_2_	RNH_2_
Starch, glucose		RNH_2_	Starch	ROH	ROH
δ^15^N		ROH	Starch, glucose	Starch	Starch
δ^13^C		Starch	δ^15^N	Starch, glucose	Starch, glucose
		Starch, glucose	δ^13^C	δ^15^N	δ^15^N
		δ^15^N		δ^13^C	δ^13^C

Statistical verification of composite signatures (Collins et al., 2014) involved the use of genetic algorithm‐driven discriminant function analysis (GA‐DFA), the Kruskal–Wallis *H*‐test (KW‐H), and principal component analysis (PCA). Table 4 presents the results of the GA‐DFA. Three alternative final composite signatures were identified for discriminating the channel bed sediment‐associated OM sources in each subcatchment. Each signature was selected on the basis of 200 repeat iterations of the GA‐DFA, using the minimization of Wilks' lambda as a stepwise selection algorithm and a probability value for parameter entry of 0.05. Final composite GA‐DFA signatures correctly classified 100% of the source samples collected in the upper River Axe, River Synderford, Blackwater River, Kit Brook, and River Yarty subcatchments and 94–100% of those source samples collected in the Temple Brook subcatchment. Tracer discriminatory weightings for the mass balance modelling for sediment‐associated OM source apportionment were estimated using the relative discrimination of the corresponding source samples by the individual properties included in each optimum composite fingerprint (Table 4).

**Table 4:** The results of the genetic algorithm‐driven discriminant function analysis (GA‐DFA) for sediment‐associated organic matter (OM) source discrimination

Upper River Axe									
GA‐DFA 1			GA‐DFA 2			GA‐DFA 3			
Property	%^a^	TDW^b^	Property	%^a^	TDW^b^	Property	%^a^		TDW^b^
ArOH	97	1.10	Aromatic	96	1.39	ArOH	97		1.41
Aromatic	96	1.09	δ^13^C	69	1.00	δ^13^C	69		1.00
CH_3_	88	1.00	CH_2_	88	1.27	CH_2_	88		1.27
HC=CH	100	1.14	CONH_2_	94	1.36	CH_3_	88		1.27
Starch, glucose	94	1.07	CONHR	100	1.45	ROH	94		1.36
Total^c^	100		Total^c^	100		Total^c^	100		

Temple Brook									
GA‐DFA 1			GA‐DFA 2			GA‐DFA 3			
Property	%^a^	TDW^b^	Property	%^a^	TDW^b^	Property	%^a^		TDW^b^
Aromatic	49	1.05	ArOH	59	1.28	HC=CH	58		1.21
CH	48	1.04	Aromatic	49	1.05	ArOH	59		1.28
CH_2_	46	1.00	δ^15^N	76	1.65	CH_2_	46		1.00
δ^15^N	76	1.65	Protein	49	1.05	δ^15^N	76		1.65
Starch, glucose	46	1.00	RNH_2_	46	1.00	ROH	51		1.11
Total^c^	94		Total^c^	100		Total^c^	100		

River Synderford									
GA‐DFA 1			GA‐DFA 2			GA‐DFA 3			
Property	%^a^	TDW^b^	Property	%^a^	TDW^b^	Property	%^a^		TDW^b^
Aromatic	73	1.59	Aromatic	73	1.59	ArNH_2_		69	1.49
C=H	69	1.49	Cellulose	46	1.00	C=H		69	1.49
Cellulose	46	1.00	CONH_2_	63	1.35	Cellulose		46	1.00
CONH	66	1.43	CONHR	66	1.43	CH_3_		60	1.30
CONH_2_	63	1.35	H_2_O	66	1.43	CONH_2_		63	1.35
CONHR	66	1.43	δ^15^N	75	1.62	CONHR		66	1.43
Protein	63	1.35	Protein	63	1.35	δ^15^N		75	1.62
Starch, glucose	61	1.32	RNH_2_	68	1.46	Protein		63	1.35
Total^c^	100		Total^c^	100		Starch, glucose		61	1.32
						Total^c^		100	

Blackwater River									
GA‐DFA 1			GA‐DFA 2			GA‐DFA 3			
Property	%^a^	TDW^b^	Property	%^a^	TDW^b^	Property	%^a^		TDW^b^
δ^13^C	94	1.14	Aromatic	85	1.00	δ^13^C	94		1.17
Cellulose	83	1.00	δ^13^C	94	1.10	Cellulose	83		1.03
CONH_2_	88	1.06	CONHR	88	1.03	CH	88		1.09
Starch	88	1.06	δ^15^N	94	1.10	RNH_2_	80		1.00
Starch, glucose	88	1.06	ROH	88	1.03	ROH	88		1.09
Total^c^	100		Total^c^	100		Total^c^	100		

Kit Brook									
GA‐DFA 1			GA‐DFA 2			GA‐DFA 3			
Property	%^a^	TDW^b^	Property	%^a^	TDW^b^	Property	%^a^		TDW^b^
CH	75	1.00	ArOH	81	1.08	Aromatic	81	1.08	
Protein	77	1.03	C=H	81	1.08	CONH	81	1.08	
CH_3_	88	1.17	CH_2_	78	1.04	CONH_2_	81	1.08	
CH_2_	78	1.04	CONH	81	1.08	HC=CH	75	1.00	
CONH	81	1.08	δ^15^N	75	1.00	ROH	81	1.08	
Total^c^	100		Total^c^	100		Total^c^	100		

River Yarty									
GA‐DFA 1			GA‐DFA 2			GA‐DFA 3			
Property	%^a^	TDW^b^	Property	%^a^	TDW^b^	Property	%^a^		TDW^b^
ArNH_2_	59	1.10	Aromatic	88	1.18	ArNH_2_	74	1.10	
Aromatic	69	1.18	CH	89	1.30	δ^13^C	69	1.03	
CONH_2_	67	1.13	CONH_2_	67	1.00	CH	87	1.30	
ROH	85	1.44	CONHR	88	1.31	HC=CH	67	1.00	
Starch, glucose	83	1.39	RNH_2_	88	1.31	δ^15^N	67	1.00	
Total^c^	100		Total^c^	100		Total^c^	100		

a % sediment‐associated OM source type samples classified correctly by individual properties.

b Tracer discriminatory weighting used in the mass balance modelling for sediment‐associated OM source apportionment.

c % sediment‐associated OM source type samples classified correctly by composite signature.

The KW‐H test was used to rank properties with the largest statistically significant differences among the OM source type pairs. The highest ranked properties were passed through the GA‐DFA to calculate the tracer discriminatory weightings and the total discriminatory efficiency of the final set of properties (Table 5). KW‐H selected final composite fingerprints correctly classified 94–100% of the samples collected to characterize sediment‐associated OM sources in the River Axe subcatchments (Table 5). Alternative composite fingerprints were identified using PCA and ranking properties with the highest loadings. Two components were consistently sufficient for explaining nearly 100% of the tracer property variance. For consistency, the individual properties and the property sets identified using the PCA were passed through the GA‐DFA to calculate tracer discriminatory weightings and to assess the percentage of the source samples classified correctly. Table 5 shows that the final fingerprints selected using PCA correctly distinguished 94–100% of the source samples.

**Table 5:** The final Kruskal–Wallis *H*‐test (KW‐H) and principal component analysis (PCA) composite signatures for sediment‐associated organic matter (OM) source discrimination

Upper River Axe					
KW‐H			PCA		
Property	%^a^	TDW^b^	Property	%^a^	TDW^b^
ArOH	97	1.71	Aromatic	96	1.71
Aromatic	96	1.71	δ^13^C	69	1.22
δ^13^C	69	1.22	CH_2_	88	1.56
CH_3_	88	1.56	HC=CH	100	1.78
δ^15^N	56	1.00	δ^15^N	56	1.00
Total^c^	100		Total^c^	100	

Temple Brook					
KW‐H			PCA		
Property	%^a^	TDW^b^	Property	%^a^	TDW^b^
ArOH	59	1.23	Aromatic	49	1.09
CH	48	1.00	CH_2_	46	1.00
HC=CH	58	1.21	Protein	49	1.09
δ^15^N	76	1.61	δ^15^N	76	1.65
ROH	51	1.08	ROH	51	1.11
Total^c^	94		Total^c^	94	

River Synderford					
KW‐H			PCA		
Property	%^a^	TDW^b^	Property	%^a^	TDW^b^
ArNH_2_	69	1.10	Aromatic	73	1.22
ArOH	64	1.02	CH	81	1.35
Aromatic	73	1.16	CH_2_	70	1.17
CH	81	1.30	CH_3_	60	1.00
CH_2_	70	1.12	CONH_2_	63	1.04
CONH_2_	63	1.00	HC=CH	73	1.21
HC=CH	73	1.16	δ^15^N	75	1.25
δ^15^N	75	1.20	Protein	63	1.04
Total^c^	100		RNH_2_	68	1.13
			Total^c^	100	

Blackwater River					
KW‐H			PCA		
Property	%^a^	TDW^b^	Property	%^a^	TDW^b^
ArOH	88	1.06	Aromatic	85	1.09
Aromatic	85	1.02	δ^13^C	94	1.21
δ^13^C	94	1.14	Cellulose	83	1.06
δ^15^N	94	1.14	CH_2_	78	1.00
Protein	83	1.00	δ^15^N	94	1.21
Total^c^	100		Total^c^	100	

Kit Brook					
KW‐H			PCA		
Property	%^a^	TDW^b^	Property	%^a^	TDW^b^
ArOH	81	1.08	δ^13^C	73	1.00
Protein	77	1.03	Cellulose	88	1.21
δ^15^N	75	1.00	CH_2_	78	1.07
CONH	81	1.08	CH_3_	88	1.21
CH	75	1.00	δ^15^N	75	1.03
Total^c^	100		Total^c^	100	

River Yarty					
KW‐H			PCA		
Property	%^a^	TDW^b^	Property	%^a^	TDW^b^
Cellulose	88	1.31	δ^13^C	69	1.03
CH_3_	88	1.31	CONH_2_	67	1.00
CONH_2_	67	1.00	HC=CH	67	1.00
HC=CH	67	1.00	δ^15^N	67	1.00
δ^15^N	67	1.00	Protein	73	1.09
Total^c^	100		Total^c^	100	

a % sediment‐associated OM source type samples classified correctly by individual properties.

b Tracer discriminatory weighting used in the mass balance modelling for sediment‐associated OM source apportionment.

c % sediment‐associated OM source type samples classified correctly by composite signature.

#### 2.1.4 Data processing for source apportionment

The relative contributions from the individual sediment‐associated OM source types were quantified using the mass balance mixing model described by Collins et al. (2014). In short, the model seeks to solve a set of linear equations for each composite signature by minimizing the sum of squares of the weighted relative errors, namely,

(3) $${\sum_{i=1}^{n}\left\{\left(C_{i}-\left(\sum_{s=1}^{m}P_{s}S_{\mathrm{si}}\text{SVsi}\right)\right)/C_{i}\right\}}^{2}W_{i},$$

where *C*_*i*_ deviates median concentration of tracer property (*i*) in the channel bed sediment samples; *P*_*s*_ is the optimized percentage contribution from source category(*s*); *S*_*si*_ deviates median concentration of tracer property (*i*) in source category(*s*); *SVsi* is the weighting representing the within‐source variation of tracer property(*i*) in source category (*s*); *W*_*i*_ is the tracer discriminatory weighting; *n* is the number of tracer properties comprising the final composite fingerprints selected using GA‐DFA, KW‐H, or PCA; *m* is the number of sediment‐associated OM source categories.

A within‐source variation weighting is incorporated into the objective function to ensure that those properties with smaller variance exert more influence on the mathematical solutions. The inverse of the coefficient of variation was used as a basis for these calculations. The tracer discriminatory power weighting in the objective function is based on the relative outputs of the discriminant function analysis for the individual properties comprising each composite fingerprint selected using GA‐DFA, KW‐H, or PCA. On this basis, the discriminatory power of the property providing the lowest discrimination (%) of the source samples in question is assigned a weighting of 1.0, and the corresponding weightings for the remainder of the properties are calculated using the ratio of their discriminatory efficiency to that of the weakest property in any specific composite signature.

Uncertainties in characterizing the median tracer values for the mass balance model on the basis of relatively few source materials and channel bed sediment samples were quantified explicitly using the scaling of the parameter distributions based on *Q*_*n*_ (Rousseeuw & Croux, 1993) and a Monte Carlo approach. Robust statistics were used because analyses using the Lilliefors test revealed that the majority of the tracers were nonuniform in distribution. Stratified repeat mixing model iterations using Latin hypercube sampling generated deviate predicted median relative source contributions. A goodness‐of‐fit test based on the absolute mean relative error (Collins, Walling, & Leeks, 1997) between source‐weighted predicted and measured sediment‐associated OM properties assessed each individual repeat solution, and the mixing model iterations continued until 5,000 viable solutions, on the basis of the goodness‐of‐fit (>0.85), were identified for each composite fingerprint for each subcatchment. Using a goodness‐of‐fit estimator based on absolute mean relative error is more robust, because widely used alternatives based on relative mean error squared can return highly acceptable fits between source‐weighted predicted and measured sediment tracer concentrations, even when the former estimator returns unacceptable fits (Laceby & Olley, 2015). The probability density functions generated by the Monte Carlo runs illustrated the full uncertainty ranges associated with the mixing model outputs and were used to estimate relative frequency‐weighted average median contributions (*R*) from the individual OM source types, namely,

(4) $$R=\sum_{i=1}^{n}v_{i}\;F_{i},$$

where *n* is the number of intervals for the predicted deviate relative contribution, scaled between 0 and 1, and *v* and *F* are the midvalue and the relative frequency for the *i*th interval, respectively.

## 3 RESULTS AND DISCUSSION

**Figure 3:**
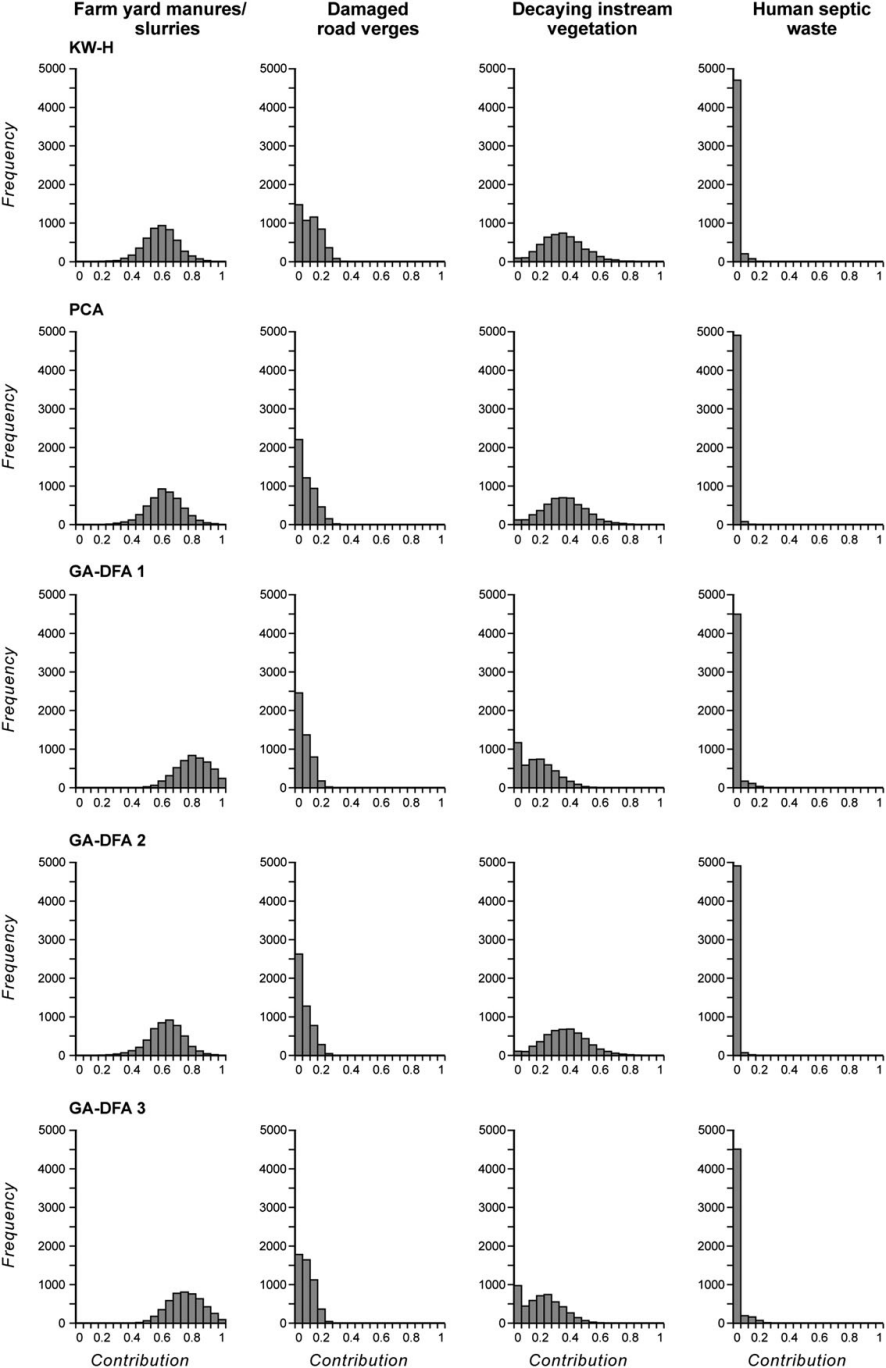
Probability density functions for the predicted deviate median relative contributions from each source type to the bed sediment‐associated organic matter collected from the Blackwater River subcatchment, using each final composite fingerprint. KW‐H = Kruskal–Wallis *H*‐test; PCA = principal component analysis; GA‐DFA = genetic algorithm‐driven discriminant function analysis

As an example, Figure 3 presents the mixing model output probability density functions for the Blackwater River subcatchment. These and the corresponding outputs for the remaining subcatchments were used as a basis for estimating relative frequency‐weighted median source contributions on the basis of each of the five composite signatures identified for each subcatchment. The final source apportionment estimates (Table 6) .were generated using a weighting combining the goodness‐of‐fit and % discriminatory power afforded by each composite signature within the set of five for any individual subcatchment. Table 6 shows that farmyard manures or slurries represented a key source of sediment‐associated OM collected across the River Axe catchment by the reconnaissance survey, with the estimated contributions ranging from 19% (uncertainty range 0–82%) in the River Synderford subcatchment to 64% (uncertainty range 0–99%) in the Blackwater River subcatchment. A corresponding range of 26% (uncertainty range 0–100%) to 44% (uncertainty range 0–100%) was recently reported by Collins et al. (2014). This source category therefore warrants further investigation to identify problem yards, positioning of heaps, or spreading practices. Findings from the reconnaissance survey suggest that the Blackwater River, the upper Axe, and Kit Brook subcatchments (Figure 1).are priorities with respect to investigating issues associated with losses of farm manures or slurries to the river channel system. Across the subcatchments, the overall average median contribution from farmyard manures or slurries to bed sediment**‐**associated OM collected by the reconnaissance survey was 36%.

**Table 6:** Relative frequency‐weighted average median source type contributions to the sediment‐associated OM sampled in the River Axe subcatchments, using each final signature

Subcatchment	Signature	Farmyard manures or slurries	Damaged road verges	Decaying instream vegetation	Human septic waste point source discharges
Upper River Axe	KW‐H	0.29	0.13	0.54	0.0**4**
	PCA	0.29	0.14	0.54	0.03
	GA‐DFA 1	0.5**4**	0.07	0.35	0.04
	GA‐DFA 2	0.67	0.14	0.15	0.04
	GA‐DFA 3	0.67	0.12	0.15	0.06
Overall weighted average^a^		0.49	0.12	0.35	0.04
Temple Brook	KW‐H	0.24	0.06	0.67	0.03
	PCA	0.25	0.04	0.69	0.02
	GA‐DFA 1	0.24	0.05	0.69	0.02
	GA‐DFA 2	0.25	0.04	0.69	0.02
	GA‐DFA 3	0.24	0.05	0.68	0.03
Overall weighted average^a^		0.25	0.05	0.68	0.02
River Synderford	KW‐H	0.14	0.13	0.70	0.0**3**
	PCA	0.1**5**	0.13	0.69	0.03
	GA‐DFA 1	0.3**0**	0.10	0.57	0.03
	GA‐DFA 2	0.19	0.07	0.7**1**	0.03
	GA‐DFA 3	0.18	0.07	0.7**2**	0.03
Overall weighted average^a^		0.19	0.10	0.68	0.03
Blackwater River	KW‐H	0.55	0.10	0.32	0.03
	PCA	0.58	0.07	0.32	0.03
	GA‐DFA 1	0.76	0.06	0.15	0.03
	GA‐DFA 2	0.58	0.06	0.33	0.03
	GA‐DFA 3	0.7**2**	0.07	0.18	0.03
Overall weighted average^a^		0.64	0.07	0.26	0.03
Kit Brook	KW‐H	0.14	0.03	0.81	0.02
	PCA	0.14	0.07	0.77	0.02
	GA‐DFA 1	0.62	0.05	0.29	0.04
	GA‐DFA 2	0.14	0.03	0.81	0.02
	GA‐DFA 3	0.59	0.04	0.34	0.03
Overall weighted average^a^		0.33	0.04	0.60	0.03
River Yarty	KW‐H	0.29	0.2**5**	0.38	0.08
	PCA	0.31	0.42	0.22	0.05
	GA‐DFA 1	0.27	0.29	0.3**7**	0.07
	GA‐DFA 2	0.29	0.30	0.3**3**	0.08
	GA‐DFA 3	0.21	0.47	0.27	0.05
Overall weighted average^a^		0.27	0.35	0.32	0.06

*Note*. OM = organic matter; KW‐H = Kruskal–Wallis *H*‐test; PCA = principal component analysis; GA‐DFA = genetic algorithm‐driven discriminant function analysis.

The bold emphasis was suggested in the author version of the paper to assist readers in extracting the overall weighted average source proportions.

a Estimated using a weighting combining the corresponding goodness‐of‐fit and % discriminatory power.

Damaged road verge contributions were estimated to be highest (35%) in the River Yarty subcatchment. Elsewhere, the contributions were typically up to ~10%. Across the study areas as a whole, the estimated average median contribution from damaged road verges to bed sediment‐associated OM was 12%. Previous work by Collins et al. (2014) also highlighted damaged road verges as an important source of sediment‐associated OM ingressing river substrates, reporting a range of 11% (uncertainty range 0–75%) to 48% (uncertainty range 0–99%). Road verges are frequently damaged by vehicle traffic and livestock movements, and the sediment‐associated OM is easily delivered to streams along metalled road and associated drain networks. Recent experimental work has demonstrated the detrimental impact of damaged road verge sediment on Atlantic salmon and brown trout in terms of sublethal impacts, reflecting the high OM content of such source material (Sear et al., 2016). The results from the reconnaissance survey herein suggest that damaged road verges are highly likely contributing to the unfavourable condition of the River Axe SAC, meaning that appropriate subcatchment‐wide solutions warrant investigation.

Decaying instream vegetation also represented an important source of bed sediment‐associated OM with the estimated contributions ranging from 22% (uncertainty range 0–100%) in the River Yarty subcatchment to 68% in both the Temple Brook (uncertainty range 0–100%) and River Synderford (uncertainty ranges 0–100%) subcatchments. For the study areas as a whole, this source category was estimated to contribute 48%. Increased shear stress in conjunction with higher flows can mobilize in‐channel decaying vegetation, and evidence has demonstrated that some of this material can infiltrate river substrates thereby contributing to oxygen consumption via subsequent decomposition (Bateman, 2012; Soulsby, Malcolm, Tetzlaff, & Youngson, 2009; Soulsby, Malcolm, & Youngson, 2001).

The reconnaissance survey suggested that human septic waste does contribute to bed sediment‐associated OM sampled across the River Axe study catchment, although this is consistently the least important source (contributions typically <5%). Septic tank discharges pose a risk to rural water quality across the UK with common reasons for failure including poor maintenance, irregular cleaning out, and the fact that many tanks are inefficient because they are >25 years old (cf. Beal, Gardner, & Menzies, 2005; May, Place, O'Malley, & Spears, 2011). Previous work by Collins et al. (2014) reported a corresponding range of 4% (uncertainty range 0–31%) to 10% (uncertainty range (0–44%).

The source apportionment results must be interpreted in the context of a number of limitations. Overall, the full uncertainty ranges around the final estimates of median source contributions suggested that inputs from farmyard manures or slurries, damaged road verges, and decaying instream vegetation are more uncertain than those from the human septic waste source category. The source sample numbers collected by any tracing investigation are inevitably constrained by available budgets and rarely, if ever, satisfy statistically based probability sampling. The collection of subsamples that are bulked into composite samples for laboratory analysis improves representativeness by taking account of microscale spatial variations, whereas the dispersal of sampling locations across a given subcatchment captures macroscale spatial variations. It is assumed that any tracer property transformation during transit to, and through, the river channel system is not significant enough to impact on the predicted source proportions. Although tracer properties are screened for significant transformation using the range test, this does not confirm a complete absence of tracer transformation. River sediment was collected from a single location towards the downstream end of each subcatchment. The estimated source proportions therefore relate to these sampling points. Source estimates are scale dependent in that they can differ for different sampling locations along a channel network as the mixture of potential sources and their connectivity to the channel system vary longitudinally (Koiter et al., 2013). An important limitation of the reconnaissance survey was its short duration. Capturing temporal variability in source contributions by sampling river sediment for longer is important as seasonal variations can exist in conjunction with land management activities, for example, the spreading of farm manures or slurries in the spring. Further discussion is provided in SI.

Table 7 compares the SOD data (0–5 and 0–20 days) for the River Axe SAC with those assembled for other rivers (Collins et al., 2015). This comparison suggests that the SOD_5_ of the channel bed sediment deposited in the River Axe SAC is joint third highest, out of the 10 rivers for which such data are presented. The SOD_20_ of the channel bed sediment deposited in the River Axe SAC is sixth highest out of the 10 rivers. SOD values for the different rivers show appreciable variability. Reasons for this are likely to be a function of the dominant type of OM (Lundkvist, Grue, Friend, & Flindt, 2007; Tank, Rosi‐Marshall, Griffiths, Entrekin, & Stephen, 2010), the quantity of OM (Thomann & Mueller, 1987), and the surface area of the particles sampled (House, 2003). Each of these factors might be expected to vary with catchment type. The River Blackwater (New Forest) catchment, for instance, is a seminatural largely forested stream. SOD in this stream is low, as might be expected in the absence of significant human impacts through widespread intensive agriculture or inputs from domestic or industrial point sources. Similarly, large parts of the River Lod subcatchment of the Western Rother River, in West Sussex, are forested, and recent unpublished source apportionment work in that area has underscored the significance of sediment inputs from forestry (relative frequency‐weighted average median contribution of 47% for contemporary channel bed sediment samples). Particulate OM derived from wood/forest or leaf sources typically has a lower Biological Oxygen Demand (BOD) as a result of the higher concentrations of refractory C in lignin (Ward, 1986). In contrast, chalk catchments, such as the rivers Test and Frome, have the highest instream biomass as a result of stable thermal and flow regimes, and the SOD values for these streams are higher. The simple comparison of SOD data provided in Table 7 illustrates how the reconnaissance survey generated medium values, suggesting that the oxygen consumption by deposited sediment along the SAC is reasonably significant in the context of corresponding information for alternative rivers.

**Table 7:** Comparison of average SOD values for the River Axe SAC with those assembled by the authors for other rivers across England and Wales

River	SOD_5_ (mg O_2_ g^−1^ dry sediment)	SOD_20_ (mg O_2_ g^−1^ dry sediment)
Axe SAC	4	7
Camel valley SSSI	15	30
Lod	1	2
Lugg	1	4
Blackwater (New Forest)	1	4
Ithon	2	6
Test	3	10
Aran	4	13
Frome	5	25
Tywi	5	13

*Note*. It should be noted that the sampling dates were not consistent across the above sites. SOD = sediment oxygen demand; SAC = Special Area of Conservation; SSSI = Site of Special Scientific Interest.

The SOD data from the River Axe SAC reported here are the products of a single sampling campaign. The SOD measurements were undertaken in the laboratory in line with previous studies, as opposed to in situ. Although it was not possible to collect repeat samples given the short duration of the reconnaissance survey, the values shown are representative of a broader range of UK rivers. The methodology demonstrates a novel approach to identifying OM sources and SOD as a reconnaissance tool for supporting more detailed targeting for source control. We conclude that the reconnaissance survey for OM sources and SOD should be extended with sample collection linked to critical periods of juvenile fish development and specific subcatchments, depending on local priorities, to improve the robustness of the dataset.

## Supporting information

Figure S1: Typical damaged road verges on a slope leading directly to a bridge crossing the channel network.

## References

[rra3175-bib-0001] Bateman, S. J . (2012). Sources and impacts of inorganic and organic fine sediment in salmonid spawning gravels in chalk rivers, Unpublished PhD Thesis, Geography & Environment, University of Southampton, p. 368.

[rra3175-bib-0002] Beal, C. D. , Gardner, E. A. , & Menzies, N. W. (2005). Process, performance, and pollution potential: A review of septic tank–soil absorption systems. Australian Journal of Soil Research, 43, 781–802.

[rra3175-bib-0003] Chevalier, B. C. , Carson, C. , & Miller W. J. (1984). Report of engineering and biological literature pertaining to the aquatic environment: With special emphasis on dissolved oxygen and sediment effects on salmonid habitat. Colorado State University, Dept. Agr. And Chem. Eng., ARS Project No. 5602‐20813‐008A.

[rra3175-bib-0004] Collins, A. L. , Jones, J. I. , Sear, D. A. , Naden, P. S. , Skirvin, D. , Zhang, Y. S. , … Hill, C. (2015). Extending the evidence base on the ecological impacts of fine sediment and developing a framework for targeting mitigation of agricultural sediment losses. Final report to Defra, September 2015.

[rra3175-bib-0005] Collins, A. L. , Naden, P. S. , Sear, D. A. , Jones, J. I. , Foster, I. D. L. , & Morrow, K. (2011). Sediment targets for informing river catchment management: International experience and prospects. Hydrological Processes, 25, 2112–2129.

[rra3175-bib-0006] Collins, A. L. , Pulley, S. , Foster, I. D. L. , Gellis, A. , Porto, P. , & Horowitz, A. J. (in press). Sediment source fingerprinting as an aid to catchment management: A review of the current state of knowledge and a methodological decision‐tree for end‐users. Journal of Environmental Management. 10.1016/j.jenvman.2016.09.075 27743830

[rra3175-bib-0007] Collins, A. L. , Walling, D. E. , & Leeks, G. J. L. (1997). Source type ascription for fluvial suspended sediment based on a quantitative composite fingerprinting technique. Catena, 29, 1–27.

[rra3175-bib-0008] Collins, A. L. , Williams, L. J. , Zhang, Y. S. , Marius, M. , Dungait, J. A. J. , Smallman, D. J. , … Naden, P. S. (2014). Sources of sediment‐bound organic matter infiltrating spawning gravels during the incubation and emergence life stages of salmonids. Agriculture, Ecosystems and Environment, 196, 76–93.

[rra3175-bib-0009] Collins, A. L. , Zhang, Y. , Walling, D. E. , Grenfell, S. E. , Smith, P. , Grischeff, J. , … Brogden, D. (2012). Quantifying fine‐grained sediment sources in the River Axe catchment, southwest England: Application of a Monte Carlo numerical modelling framework incorporating local and genetic algorithm optimisation. Hydrological Processes, 26, 1962–1983.

[rra3175-bib-0010] Comber, A. , Anthony, A. , & Proctor, C. (2008). The creation of a national agricultural land use dataset: Combining pycnophylactic interpolation with dasymetric mapping techniques. Transactions in GIS, 12, 775–791.

[rra3175-bib-0011] Cooper, R. J. , Pedentchouk, N. , Hiscock, K. M. , Disdle, P. , Krueger, T. , & Rawlins, B. G. (2015). Apportioning sources of organic matter in streambed sediments: An integrated molecular and compound‐specific stable isotope approach. Science of the Total Environment, 520, 187–197. 25817221 10.1016/j.scitotenv.2015.03.058

[rra3175-bib-0012] Delzer, G. C. , & McKenzie, S. W. (2003). Five‐day biochemical oxygen demand. US Geological Survey Techniques of Water‐Resources Investigations, book 9, chapter A7, 3^rd^ edition.

[rra3175-bib-0013] Doyle, M. C. , & Lynch, D. D. (2005). Sediment oxygen demand in Lake Ewauna and the Klamath River, Oregon, June 2003. U.S. Geological Survey Scientific Investigations Report 05‐5228, p. 14.

[rra3175-bib-0014] Duerdoth, C. P. , Arnold, A. , Murphy, J. F. , Naden, P. S. , Scarlett, P. , Collins, A. L. , … Jones, J. I. (2015). Assessment of a rapid method for quantitative reach‐scale estimates of deposited fine sediment in rivers. Geomorphology, 230, 37–50.

[rra3175-bib-0015] Greig, S. M. , Sear, D. A. , Smallman, D. J. , & Carling, P. A. (2005). Impact of clay particles on the cutaneous exchange of oxygen across the chorion of Atlantic salmon eggs. Journal of Fish Biology, 66, 1681–1691.

[rra3175-bib-0035] House, W. A. (2003). Geochemical cycling of phosphorus in rivers. Applied Geochemistry, 18, 739–748.

[rra3175-bib-0016] Kemp, P. , Sear, D. , Collins, A. , Naden, P. , & Jones, I. (2011). The impacts of fine sediment on riverine fish. Hydrological Processes, 25, 1800–1821.

[rra3175-bib-0017] Koiter, A. J. , Lobb, D. A. , Owens, P. N. , Petticrew, E. L. , Tiessen, K. H. D. , & Li, S. (2013). Investigating the role of connectivity and scale in assessing the sources of sediment in an agricultural watershed in the Canadian prairies using sediment source fingerprinting. Journal of Soils and Sediments, 13, 1676–1691.

[rra3175-bib-0018] Laceby, J. P. , & Olley, J. (2015). An examination of geochemical modelling approaches to tracing sediment sources incorporating distribution mixing and elemental correlations. Hydrological Processes, 29, 1669–1685.

[rra3175-bib-0019] Lambert, C. P. , & Walling, D. E. (1988). Measurement of channel storage of suspended sediment in a gravel‐bed river. Catena, 15, 65–80.

[rra3175-bib-0020] Lundkvist, M. , Grue, M. , Friend, P. L. , & Flindt, M. R. (2007). The relative contributions of physical and microbiological factors to cohesive sediment stability. Continental Shelf Research, 27, 1143–1152.

[rra3175-bib-0021] May L , Place C , O'Malley MO , Spears B . 2011 The impact of phosphorus inputs from small discharges on designated freshwater sites. Final report to Natural England and the Broads Authority, p. 130.

[rra3175-bib-0022] Naden, P. S. , Murphy, J. S. , Old, G. H. , Newman, J. , Scarlett, P. , Harman, M. , … Jones, J. I. (2016). Understanding the controls on deposited fine sediment in the streams of agricultural catchments. Science of the Total Environment, 347, 366–381. 10.1016/j.scitotenv.2015.12.079 26789373

[rra3175-bib-0023] Rousseeuw, P. J. , & Croux, C. (1993). Alternatives to the median absolute deviation. Journal of the American Statistical Association, 88, 1273–1283.

[rra3175-bib-0024] Sear, D. A. , Frostick, L. B. , Rollinson, G. , & Lisle, T. E. (2008). The significance and mechanics of fine sediment infiltration and accumulation in gravel spawning beds In Sear D. A., & DeVries P. D. (Eds.), Salmonid spawning habitat in rivers: Physical controls, biological responses, and approaches to remediation (pp. 149–174). Bethesda, USA: American Fisheries Society.

[rra3175-bib-0025] Sear, D. A. , Jones, J. I. , Collins, A. L. , Hulin, A. , Burke, N. , Bateman, S. , … Naden, P. S. (2016). Does fine sediment source as well as quantity affect salmonid embryo mortality and development? Science of the Total Environment, 541, 957–968. 26473698 10.1016/j.scitotenv.2015.09.155

[rra3175-bib-0026] Sear, D. A. , Pattison, I. , Collins, A. L. , Newson, M. D. , Jones, J. I. , Naden, P. S. , & Carling, P. A. (2014). Factors controlling the temporal variability in dissolved oxygen regime of salmon spawning gravels. Hydrological Processes, 28, 86–103.

[rra3175-bib-0027] Soulsby, C. , Malcolm, I. A. , Tetzlaff, D. , & Youngson, A. F. (2009). Seasonal and inter‐annual variability in hyporheic water quality revealed by continuous monitoring in a salmon spawning stream. River Research and Applications, 10, 1304–1319.

[rra3175-bib-0028] Soulsby, C. , Malcolm, I. A. , & Youngson, A. F. (2001). The hydrochemistry of the hyporheic zone in salmon spawning gravels: A preliminary assessment in a small regulated stream. Regulated Rivers, 17, 651–665.

[rra3175-bib-0029] Tank, J. L. , Rosi‐Marshall, E. J. , Griffiths, N. A. , Entrekin, S. A. , & Stephen, M. L. (2010). A review of allochthonous organic matter dynamics and metabolism in streams. Journal of the North American Benthological Society, 29, 118–146.

[rra3175-bib-0030] Thomann, R. V. , & Mueller, J. A. (1987). Principles of surface water quality modeling and control (pp. 488). Harper and Row, New York, NY: Harper International Edition.

[rra3175-bib-0031] Walling, D. E. (2013). The evolution of sediment source fingerprinting investigations in fluvial systems. Journal of Soils and Sediments, 1310, 1658–1675.

[rra3175-bib-0032] Walling, D. E. , Collins, A. L. , & McMellin, G. (2003). A reconnaissance survey of the source of interstitial fine sediment recovered from salmonid spawning gravels in England and Wales. Hydrobiologia, 497, 91–108.

[rra3175-bib-0033] Walling, D. E. , & Foster, I. D. L. (2016). Using environmental radionuclides and sediment geochemistry for tracing and dating fine fluvial sediment In Kondolf G. M., & Piégay H. (Eds.), Tools in fluvial geomorphology (2nd ed.) (pp. 183–209). Chichester: Wiley.

[rra3175-bib-0034] Ward, G. M. (1986). Lignin and cellulose content of benthic particulate organic matter (FPOM) in Oregon Cascade Mountain streams. Journal of the North American Benthological Society, 5, 127–139.

