# Supplementary material for: Sediment‐associated organic matter sources and sediment oxygen demand in a Special Area of Conservation (SAC): A case study of the River Axe, UK
Source: River Res Appl. 2017 Jun 29;33(10):1539–52. doi: 10.1002/rra.3175 (PMC5832314; doi:10.1002/rra.3175)
Supplement: Supplementary file 1 — Figure S1: Typical damaged road verges on a slope leading directly to a bridge crossing the channel network. [file RRA-33-1539-s001.docx]

**Supplementary information**

**Connectivity of the source material sampling locations**

Sampling sites representative of each source category and with good connectivity to the river channel network is a fundamental principle of the source tracing approach. The sampling of farm yard manures/slurries included fresh excreta from yards, steadings and housings, as well as waste material from dry or wet stores and manure heaps in fields. Yards, steadings, housing and wet/dry stores were frequently connected to the channel network by farm tracks, although in some cases, these potential sources were located adjacent to streams. Manure heap sampling focused on those heaps which were proximal to the river channel. Damaged road verges (Figure S1) were connected to the channel network on the basis of sloping roads leading directly to river crossings (e.g. Figure S1) or by road drains. Connectivity was not an issue for decaying instream vegetation since this was sampled from within the channel network. Some septic tanks had pipes leading directly into the stream network, whilst others were connected via drainage field biomats.


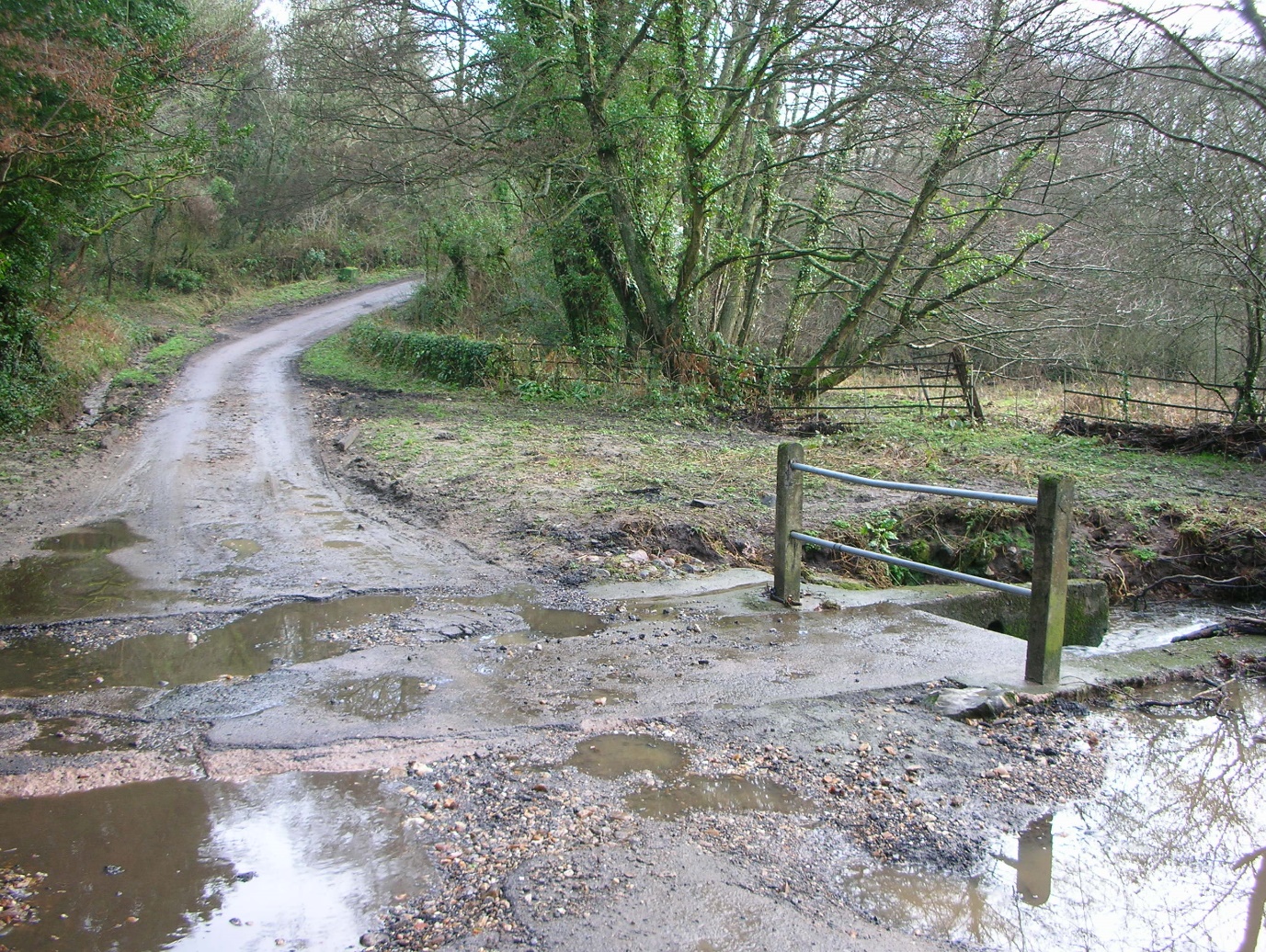


Figure S1: Typical damaged road verges on a slope leading directly to a bridge crossing the channel network.

**The timing of catchment sampling in the context of agricultural and natural processes**

Capturing temporal variability in source contributions by sampling river sediment for longer is advisable since seasonal variations can exist in conjunction with both land management activities and natural processes. In relation to the former, for example, the spreading of farm manures/slurries in the spring could logically be expected to be associated with increased contributions from this source category. Similarly, natural, vegetation decay will be higher in autumn and winter and therefore, sediment sampling during these seasons could be expected to be associated with higher contributions of sediment-associated OM from the damaged road verge and decaying instream vegetation source categories. Conceptually, human septic waste contributions could be expected to be relatively higher during the summer period, when inputs from more rainfall-dependent sources would be lower.

**Sediment sampling in the river channel system**

River channel sediment sampling for the sourcing exercise targeted, where possible, the pool-riffle morphology characteristic of the study catchment channel network. At each location, a riffle and pool habitat were sampled to capture variability in the composition of fine-grained sediment ‘patches’ associated with contrasting river flow velocity conditions. Riffle environments are used for spawning habitat by the lithophilous fish species found in the study catchment on account of the surface water – hyporheic water exchanges. Sampling of pools where fine-grained sediment retention was higher, ensured collection of sufficient sample mass for laboratory analyses of either fingerprint properties or sediment oxygen demand (SOD). In combination, the sampling of riffles and pools ensured collection of fine-grained sediment from the main meso-habitats along the channel network. Where the bed is heavily armoured, an iron bar can be used within the stilling well to assist bed disruption and re-suspension of interstitial sediment. In-stream vegetation patterns were not taken into account, given that sediment sample collection occurred during the winter period and therefore before growth in the spring and summer months.
